# Evaluating the Impact of the Tamm–Dancoff Approximation on X-ray Spectrum Calculations

**DOI:** 10.1021/acs.jctc.3c01341

**Published:** 2024-02-22

**Authors:** Thomas Fransson, Lars G. M. Pettersson

**Affiliations:** Department of Physics, AlbaNova University Center, Stockholm University, 109 61 Stockholm, Sweden

## Abstract

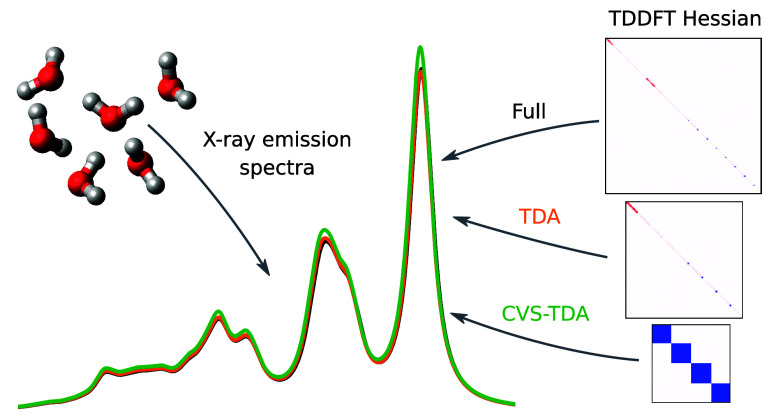

The impact of the Tamm–Dancoff approximation (TDA) for time-dependent density functional theory (TDDFT) calculations of X-ray absorption and X-ray emission spectra (XAS and XES) is investigated, showing small discrepancies in the excitation energies and intensities. Through explicit diagonalization of the TDDFT Hessian, XES was considered by using full TDDFT with a core-hole reference state. This has previously not been possible with most TDDFT implementations as a result of the presence of negative eigenvalues. Furthermore, a core–valence separation (CVS) scheme for XES is presented, in which only elements including the core-hole are considered, resulting in a small Hessian with the dimension of the number of remaining occupied orbitals of the same spin as the core-hole (CH). The resulting spectra are in surprisingly good agreement with the full-space counterpart, illustrating the weak coupling between the valence–valence and valence–CH transitions. Complications resulting from contributions from the discretized continuum are discussed, which can occur for TDDFT calculations of XAS and XES and for TDA calculations of XAS. In conclusion, we recommend that TDA be used when calculating X-ray emission spectra, and either CVS-TDA or CVS-TDDFT can be used for X-ray absorption spectra.

## Introduction

For the investigation of the electronic and atomic structure of molecular materials, X-ray spectroscopies provide a number of element-specific probes, capable of investigating local structure, occupied states, unoccupied states, and more.^1−5^ Included here are X-ray absorption spectroscopy (XAS), which probes the absorption cross-section of the system and yields insight into unoccupied states and local structure (depending on if the region around or well above the core-ionization energy is measured), and X-ray emission spectroscopy (XES), for which the fluorescence decay of core-ionized or core-excited molecules provides insight into occupied states. As a result of the large differences between core-orbital energies of different elements, in particular for the inner core region, these techniques are element-specific and capable of investigating both the local electronic and atomic structure.

Modeling X-ray spectra is relatively challenging due to effects such as strong relaxation resulting from the creation/annihilation of a core-hole, strong relativistic effects, significant self-interaction errors in density functional theory (DFT), and more. For X-ray absorption spectra, the sought core-excitations are embedded in a continuum of valence transitions, and several different approaches in which this complication can be circumvented are available. One such approximate approach is the transition-potential method with a half-occupied core level and considering only transitions between this core-orbital and the unoccupied states.^6,7^ Focusing on methods based on linear response theory, the weak coupling between core- and valence-excited states can be exploited by neglecting valence-excited terms in the excitation manifold, leading to the core–valence separation (CVS) scheme.^8,9^ This approach has been implemented in methods such as time-dependent Hartree–Fock and DFT (TDHF and TDDFT),^10−14^ equation-of-motion coupled cluster theory (primarily EOM-CCSD),^15−17^ and the algebraic-diagrammatic construction (ADC) scheme for the polarization propagator^8,9,18−20^ and has been demonstrated to only yield small errors.^21^ Additional approaches for calculating XAS using linear response theory are available, such as energy-specific approaches,^22^ the Lanczos-chain method,^23,24^ and damped response theory.^25^ For X-ray emission spectra, an approach which uses ground state orbitals has been successfully applied,^12,13,26−30^ in which energies are estimated from orbital energy differences, and intensities from transition moments between these orbitals. However, this approach yields poor absolute energies, and issues related to delocalized MOs can be present.^31^ Alternatively, it is possible to model X-ray emission processes by constructing a core-ionized reference state^32−34^ and apply linear response theory on top of such a state.^11^ Excitations into this core-hole then appear as negative eigenvalues and have been calculated using methods such as TDDFT,^29,12,30,31,35−37^ EOM-CCSD,^11,35,31,38^ and ADC.^31,39,40^ This gives access to the transition energies, intensities, and excited state properties for all transitions to this core-hole in a single calculation; in the case of more than one equivalent center each relevant core-hole is considered in turn.

Focusing on the use of linear-response TDDFT, low computational cost is achieved, but issues of insufficient relaxation and self-interaction plague X-ray spectrum calculations and result in substantial inaccuracies in absolute energies.^4,11,35,29,41,42^ This is partially due to spurious self-interactions in the final state, with the occupied core state being too high in energy for XAS (underestimating energy differences), while the unoccupied core-level is too low in energy for XES (overestimating energy differences). These inaccuracies are to some extent canceled out by the lack of relaxation effects, but most exchange-correlation functionals yield too low XAS and too high XES energies when using TDDFT. Provided that the spectrum features are reasonable, it is then possible to apply an overall shift in transition energies, and functionals tailored for use in X-ray spectroscopies have been developed (e.g., short-range corrected functionals^43^).^4^

A common approach when using TDDFT is to apply the Tamm–Dancoff approximation (TDA), in which the coupling between excitations and de-excitations is neglected.^44^ This leads to a simplified (Hermitian) problem of lower dimension, and it has been seen to yield relatively minor discrepancies in excitation energies,^44−47^ and can yield superior results for situations such as triplet instabilities.^45,47,48^ However, sum rules are no longer preserved, and there can thus be concerns regarding the quality of intensities.^45−47^ In practice, this is seldom the case, and TDA is routinely used in many applications. In fact, for XES the presence of negative eigenvalues when using a core-hole reference state prohibits most TDDFT implementations from being used,^49^ and TDA has, to the best of our knowledge, been used for all core-hole reference TDHF and TDDFT calculations to date. The impact of this approximation on the quality of calculated spectra has thus not been investigated, and we now seek to do precisely that. Furthermore, we introduce a CVS scheme for calculating XES, in which only terms including the core-hole are considered. This leads to a substantial reduction in involved matrix dimensions, but the resulting spectra involving lower core-levels are seen to be surprisingly similar to those of full TDDFT.

The present work is organized as follows: First we present a brief overview of TDDFT, TDA, and the CVS approximation, including illustrations on the involved matrices for a test case, followed by a section on computational details. In the results section, we show the impact of TDA and different CVS versions for the UV/vis, XAS, and XES spectra of gas phase water, followed by a systematic study of its effect on energies and intensities for a number of small molecules, the identification of the largest deviation for XES, and XES for a model liquid system. Finally, we discuss the impact of TDA for XAS and XES of heavier elements and outer core regions, for which complications due to intense valence-continuum states arise. These contributions are considered in some detail for calculations of X-ray absorption spectra. We note that exploratory investigations such as the present are made increasingly straightforward by leveraging the advantages of a modern, modular, and interactive computational paradigm,^50,51^ using, e.g., Jupyter Notebook^52,53^ and a workflow written in Python. This has been successfully applied in projects such as,^50^ e.g., PySCF,^54,55^ eChem,^56^ and psi4numpy.^57^

## Methodology

Within linear-response time-dependent density functional theory (TDDFT), excitation energies and transition amplitudes are obtained from the eigenvalues and eigenvectors of the non-Hermitian eigenvalue equation^58,59^
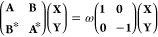
1where the left-most matrix is the electronic Hessian.^60^ For a hybrid exchange-correlation (xc) functional, the matrix elements are given as^59^
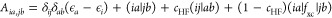
2
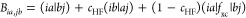
3where the two-electron integrals are given in Mulliken notation and *f*_xc_ is the exchange-correlation kernel given as the second functional derivative of the ground-state functional. The amount of Hartree–Fock exchange is *c*_HF_, such that pure TDHF or TDDFT are obtained in the limit of *c*_HF_ = 1 and *c*_HF_ = 0, respectively. Neglecting the contributions from the **B** block, i.e., diagonalizing only **A**, yields the Tamm–Dancoff approximation (TDA),^44^ in which eq 1 is simplified to
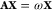
4This effectively decouples excitations and de-excitations, allowing only transitions between occupied and virtual orbital pairs, while the full formulation also allows virtual-occupied de-excitations. The computational cost is decreased (at least for hybrid xc-functionals^59^), and TDA has been seen to provide excitation energies in close agreement with the full TDDFT formalism and to remove issues related to triplet instabilities.^59,48^ However, TDA does not obey the Thomas–Kuhn–Reiche sum rule, and there are thus some concerns regarding the accuracy of intensities.^59,45,46^ For practical calculations TDA and full TDDFT typically yield very similar spectra, however.^61^ For historical reasons, the TDHF version of eq 1 is also referred to as the random-phase approximation (RPA),^59,45,47^ while the TDA counterpart corresponds to configuration interaction singles (CIS). The full TDDFT equation is sometimes also referred to as RPA,^47^ but we here chose to refer to the full-space problem as TDDFT and the reduced as TDA.

For TDA the solution of a Hermitian eigenvalue equation (eq 4) is sought, and this is commonly done by using a Davidson-like iterative scheme in which eigenstates are calculated from low (positive) eigenvalues and upward. Full TDDFT instead yields a pseudo-Hermitian eigenvalue equation, with each block being Hermitian. Assuming that the orbitals are real, it is possible to rewrite eq 1 as a Hermitian eigenvalue problem of half the dimension^59^
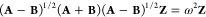
5with
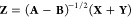
6This can be solved by using a Davidson-like iterative scheme, and many implementations of TDDFT utilize such a formalism. However, this assumes that  is positive definite,^59^ which is not the case when using a core-hole reference state. It can also be an issue for triplet (near)instabilities and if the system possesses a degenerate ground state. As such, all previous studies of XES using TDDFT with a core-hole reference state have, to the best of our knowledge, used TDA, and the impact of this approximation is unknown. In the present work, we seek to remedy this by considering full TDDFT and using explicit diagonalization of
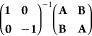
7where we assumed real orbitals. This yields eigenvalues corresponding to excitation energies and eigenvectors which are used to calculate transition dipole moments. Oscillator strengths are calculated as
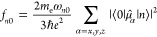
8For full TDDFT excitation energies of ±ω are obtained, while TDA yields only positive eigenvalues (for normal ground state reference states—a core-hole reference yields negative eigenvalues for transitions into the core-hole). This explicit diagonalization is typically not employed, and the scaling of this approach is high—*O*(*M*^3^) where *M* for unrestricted TDDFT is 2(*n*_occ_^α^*n*_virt_^α^ + *n*_occ_^β^*n*_virt_^β^).^59^

For X-ray absorption spectrum calculations, the sought transitions are embedded deep within the valence-continuum region, and a direct calculation of *all* states up to and including these is feasible only for small molecules. Instead, the weak coupling between the valence- and core-excitations can be leveraged by neglecting the valence-transition elements, yielding the core–valence separation (CVS) scheme.^8−10,19,15^ In eqs 2 and 3 this corresponds to removing all matrix elements except those where *i* refers to the probed core orbital(s). The core excitations are then the lowest eigenstates, and the impact of this approximation has been seen to be small.^21^ For XAS this approach has here been implemented by removing all such matrix elements and diagonalizing the CVS-versions of the TDA and TDDFT equations. Furthermore, a version of CVS for calculating X-ray emission spectra has been implemented, where only matrix elements where *a* refers to the core-hole are kept.

**Figure 1:**
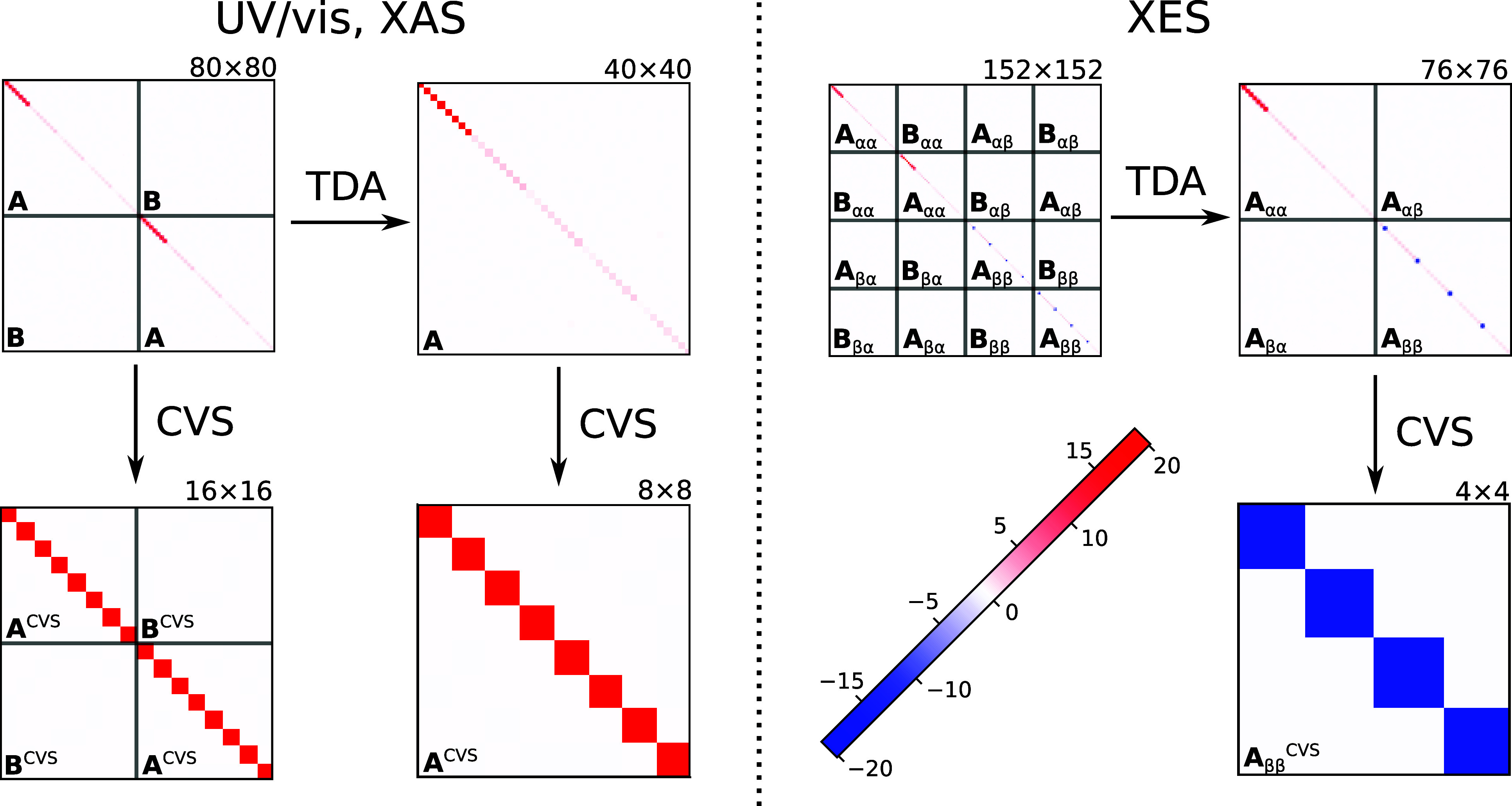
Electronic Hessians of the different TDDFT and TDA schemes, as obtained for water at the BHandHLYP/6-31G level of theory. Energies are expressed in Hartree.

In order to illustrate the different TDDFT and TDA schemes considered in this work, Figure 1 shows the resulting Hessians for gas phase water calculations at the BhandHLYP/6-31G level of theory. With this basis, there are 13 basis functions, yielding 5 occupied and 8 virtual orbitals. Restricted DFT was used for the UV/vis and XAS calculations, while the core-hole interaction in XES necessitates the use of unrestricted DFT for these spectrum calculations. For XES the total Hessian thus contains αα and ββ blocks (upper left and lower right) and the coupling blocks. Lines are included in the figure to separate the Hessians into the different blocks, and matrix dimensions are provided.

For the UV/vis and XAS calculations, the full Hessian is of dimension 2*n*_occ_*n*_virt_, which is reduced to half for TDA and to 2*n*_core_*n*_virt_ for CVS-TDDFT. For XES the core-hole has been placed in the β spin symmetry (as seen by the negative diagonal elements in the lower right block), and the full αα block remains at size 80. The ββ block is of size 2*n*_occ_^β^*n*_virt_^β^, and with a core-hole this is equal to 72. With our new CVS scheme, only terms containing a core-hole are kept, resulting in a small matrix of dimension *n*_occ_^β^.

## Computational Details

Geometries of the isolated molecules were optimized at the MP2/cc-pVTZ^62^ level of theory, using the Q-Chem 5.2 software package.^63^ Spectrum calculations were performed using the PySCF package,^54,55^ employing the BhandHLYP exchange-correlation functional,^64^ provided by the libxc library.^65^ PySCF enables direct access to objects such as Hessians and transition dipole moments, and the spectra were then calculated through direct diagonalization implemented in Python scripts and using Jupyter Notebook.^52^ Control calculations using native TDDFT and TDA solvers in PySCF were performed where possible, considering UV/vis with TDDFT and TDA, XAS with TDDFT and TDA (without CVS, so solving for many states), and TDA for XES. This reproduced the results we obtained with direct diagonalization, and we are confident in our approach. For the liquid water calculations, six water molecules were included in each cluster, using the 20 structures considered in ref (31), originally from ref (66). Unless otherwise stated, property calculations were run using a 6-311G* basis set,^67^ employing uncontracted core basis functions for the probed atom—also labeled as u6-311G*.^35,39^ Other (non-hydrogen) atoms were given an effective core potential (ECP) of the Stuttgart–Cologne type^68^ and the associated basis set. This relatively small basis set and the lack of relativistic effects (which are particularly important for 2p, as a result of the strong spin–orbit coupling with resulting peak split) are insufficient for a direct comparison to experiment. Additional diffuse functions were added for TDA and CVS-TDA calculations on H_2_Se and SF_6_, in order to probe contributions from discretized continuum states. The first exponents of these functions are 1.238 and 0.884, and this was used in an even-tempered manner to create sets ranging in size from 10s10p to 25s25p25d. For the water clusters (six molecules), only the spectrum of the central molecule was considered. Convolution of the obtained energies and intensities (oscillator strengths) using a Lorentzian broadening function was performed using a half-width at half-maximum (HWHM) of 0.3 eV, unless stated otherwise. X-ray emission spectra of 2p- and 3p-levels were constructed by performing three separate calculations with core-holes localized in each *n*p-level, and then constructing the mean spectrum.

## Results and Discussion

### Electronic Spectra of Water

**Figure 2:**
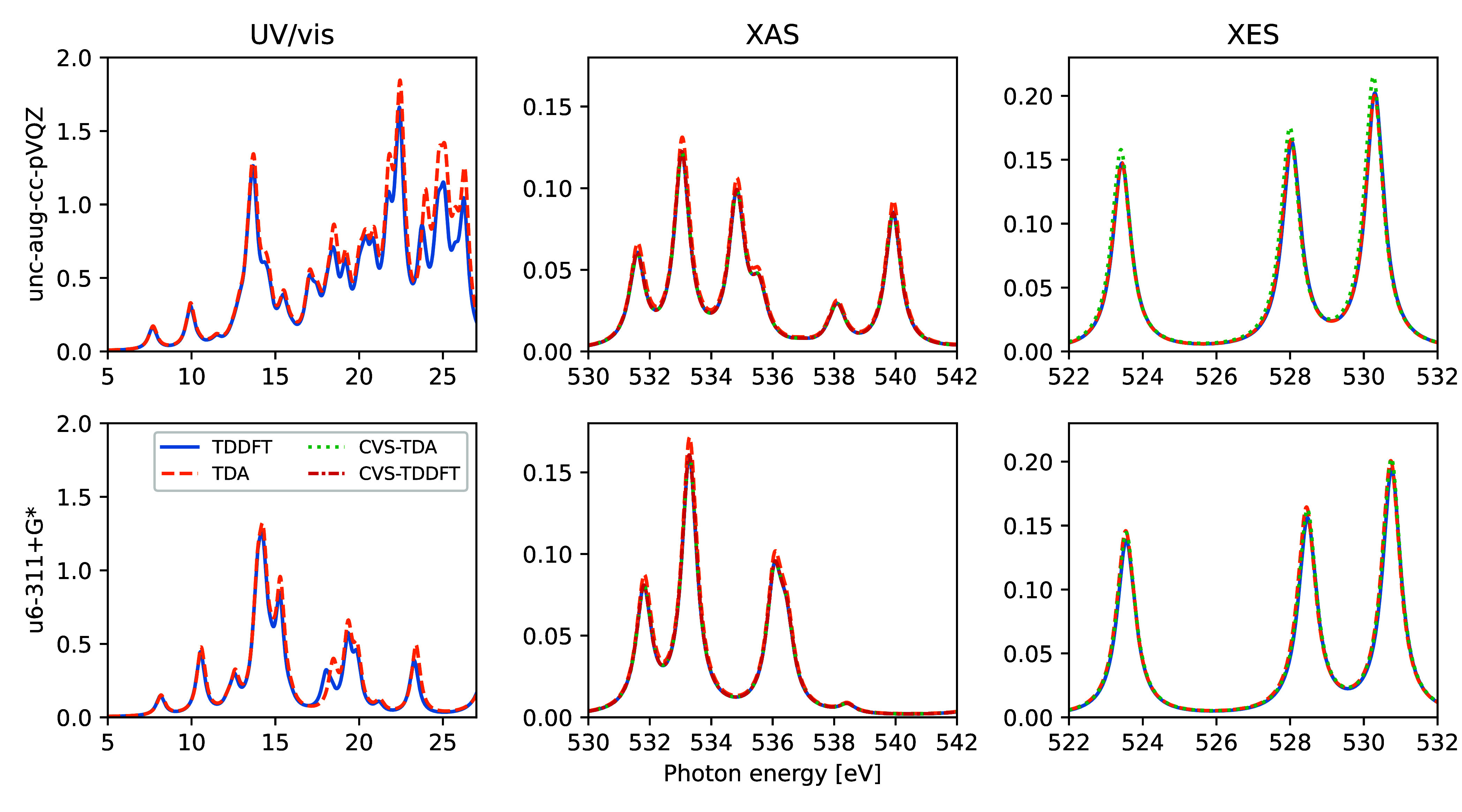
Computed UV/vis, XAS, and XES spectra of gas phase water, as obtained using different TDDFT/TDA schemes. Two basis sets were used: 6-311+G* with uncontracted core basis function (u6-311+G*, lower panels) and fully uncontracted aug-cc-pVQZ (upper panels), with a total of 33 and 189 basis functions, respectively.

Computed UV/vis, XAS, and XES spectra of gas phase water are shown in Figure 2, using the BhandHLYP xc-functional and two different basis sets. The differences in spectrum features for the different TDDFT/TDA schemes are small, primarily amounting to slight changes in intensities. Somewhat surprisingly, the CVS-TDA X-ray emission spectra are almost identical to those of TDDFT, despite a massive reduction in matrix dimensions (4 for CVS-TDA, compared to 512 and 3320 for TDDFT with the two different basis sets). Additional calculations of the X-ray emission spectra with 15 different global xc-functionals yield energy and oscillator strength discrepancies of −0.03 ± 0.03 eV and 2 ± 4% for TDA and −0.05 ± 0.04 eV and 6 ± 2% for CVS-TDA, respectively, when compared to the full TDDFT results.

### Second-Row Elements

**Figure 3:**
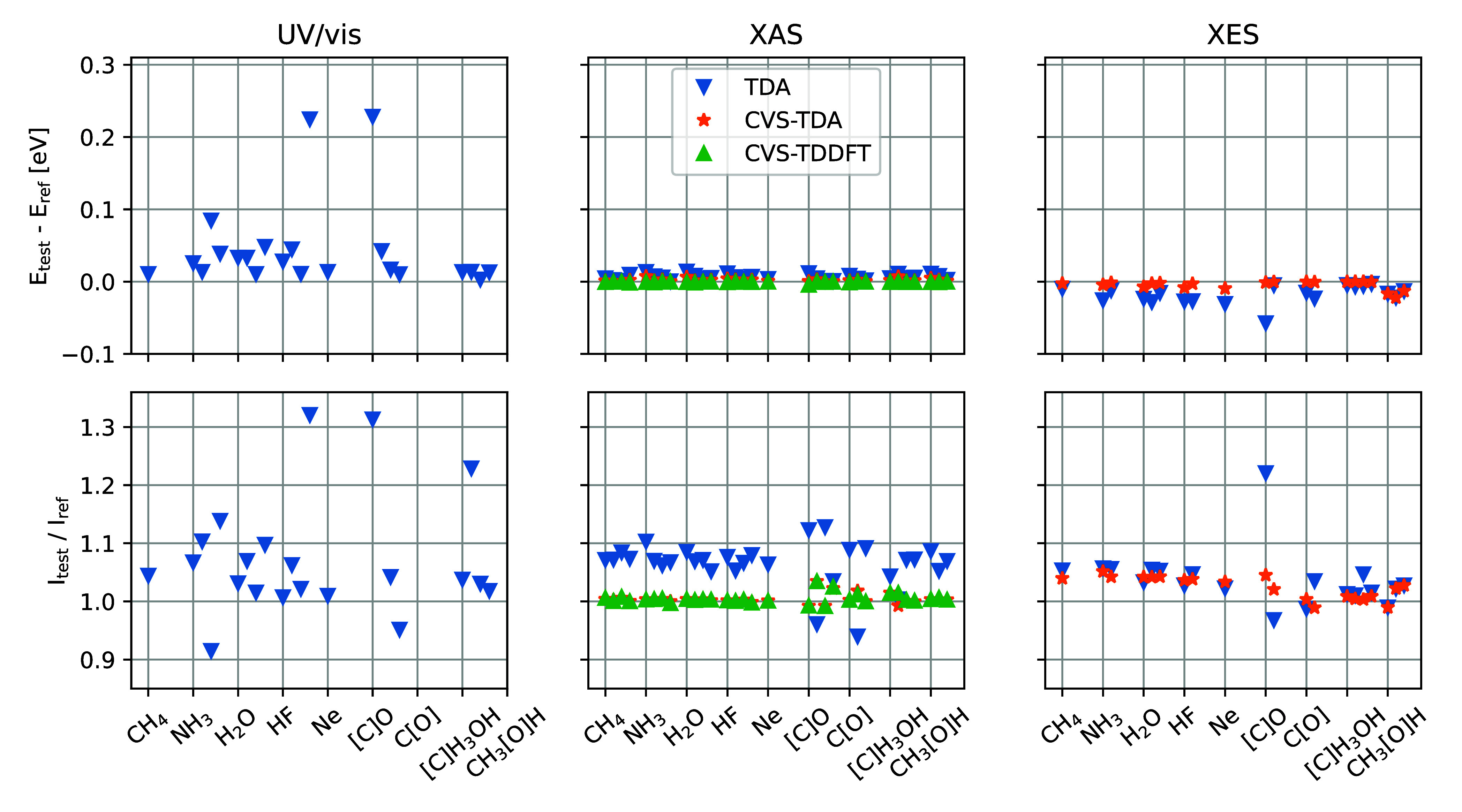
Discrepancies in transition energies and oscillator strengths for computed UV/vis, XAS, and XES spectra using TDA, CVS-TDA, and CVS-TDDFT, compared to full TDDFT.

Energy and oscillator strength discrepancies for a number of different molecules are illustrated in Figure 3, considering 1–4 transitions per molecule and the C, N, O, F, and Ne K-edges for the X-ray spectra. UV/vis spectra show the largest discrepancies, with 0.00–0.25 eV higher excitation energies for TDA, and oscillator strengths which are most often overestimated. For XAS the comparisons include CVS-TDA and CVS-TDDFT, and only very small energy differences are observed. For the oscillator strengths, there is a general tendency for TDA to yield ∼8% more intense features, while both CVS-TDA and CVS-TDDFT are close to the full TDDFT results. Finally, for XES the TDA energies are slightly lower than the reference, and the spread in oscillator strengths is about 5% for TDA and CVS-TDA, with the carbon K-edge of CO yielding outlying results for TDA. Similar trends are observed using TDHF and TDDFT with the BLYP xc-functional.

**Figure 4:**
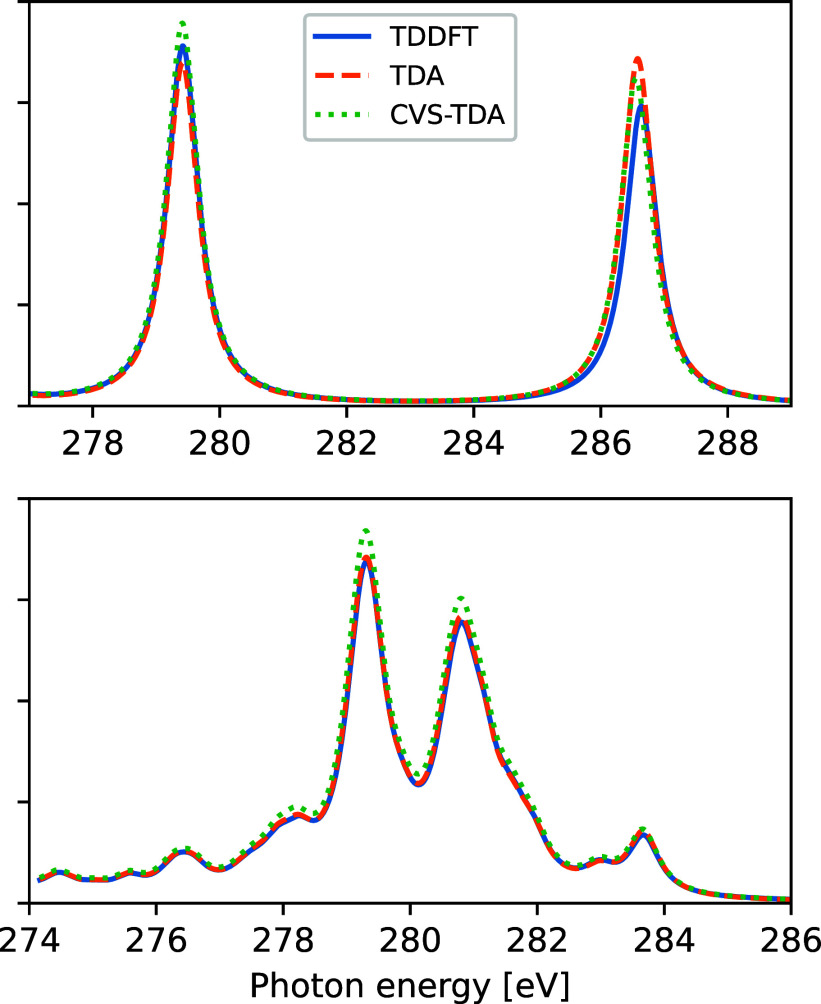
Carbon X-ray emission spectra of carbon monoxide (top) and methyl acetate (bottom), as obtained using different TDDFT/TDA schemes.

In order to further investigate the stability of TDA and CVS-TDA for XES calculations, we considered the emission spectra of all molecules in the XABOOM benchmark set,^69^ examining the K-edge of all C, N, O, and F atoms. This benchmark set consists of 40 organic (closed-shell) molecules up to the size of guanine, including different structural motifs such as unsaturated aliphatic hydrocarbons, heterocycles, aromatic hydrocarbons, carbonyl compounds, nucleobases, and more. The largest discrepancy in XES was obtained for the high-energy feature of CO, with energy and oscillator strength differences between TDA and TDDFT of −0.06 eV and 22%. These spectra are shown in Figure 4, where we also include the spectrum of one of the larger molecules which showed noticeable differences in spectrum features. The differences for the larger molecules mainly amount to small changes in absolute intensity between CVS-TDA and TDDFT/TDA. However, the majority of spectra are very similar, regardless of whether CVS-TDA, TDA, or TDDFT is used.

**Figure 5:**
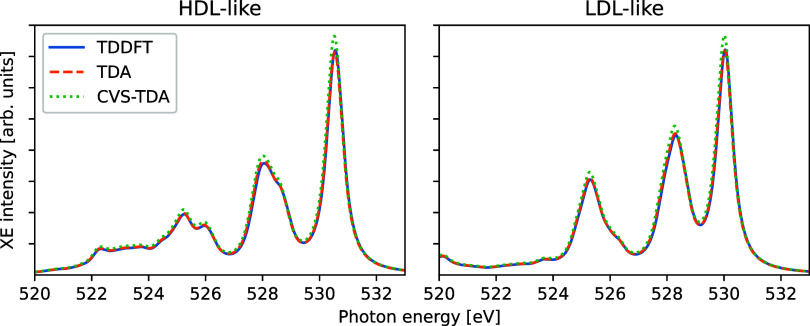
X-ray emission spectra of 10 high-density liquid (HDL) and 10 low-density liquid (LDL) water clusters, as calculated using different TDDFT/TDA schemes.

In terms of the effects of (CVS-)TDA for a model liquid, Figure 5 shows the calculated X-ray emission spectra of different water clusters, obtained from ref (31). Twenty different six-molecule clusters were considered, ten of which represent asymmetric structures (representing high-density liquid, HDL, local environment) and ten highly tetrahedral (representing low-density liquid, LDL, local environment), and the spectra were calculated for the central water molecule. The resulting differences are minor and primarily amount to a small rescaling in intensity across the spectra (in particular for CVS-TDA).

As CVS-TDA yields surprisingly good spectra, we further investigated the importance of the remaining coupling elements in the Hessian by performing calculations where only the diagonal elements in CVS-TDA are kept. For gas phase water this removes elements with a total absolute sum of 0.062 hartree, or <0.1% of the absolute sum of the Hessian, and the final spectrum is close to full CVS-TDA. For the clusters this neglects more coupling, amounting to an absolute sum of 1.69–4.02 hartree, or 0.4–0.9% of the total absolute sum. The resulting spectra are substantially distorted (results not shown), and the amount of discrepancy approximately scales with the absolute sum of the ignored matrix elements. This difference between the gas and cluster results reflects the stronger valence–valence couplings in the latter, resulting in increasingly poor spectra. However, the full CVS-TDA approach is seen to work well for both the isolated molecule and the clusters, as the coupling between the core-hole and valence is weak for all these systems.

### Heavier Elements and Outer Core Levels

**Figure 6:**
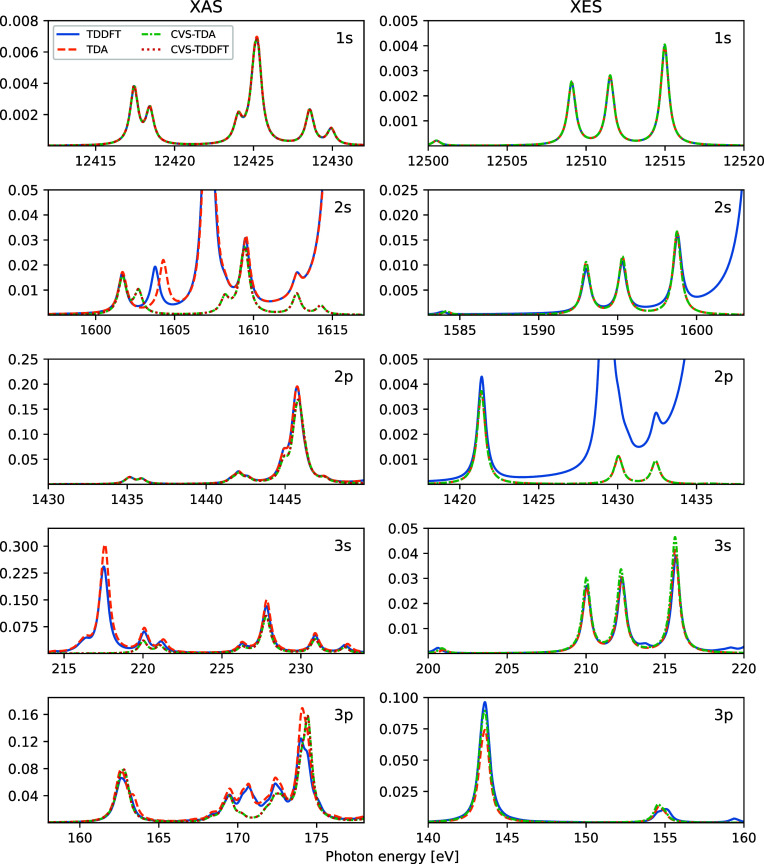
X-ray absorption and emission spectra of hydrogen selenide (H_2_Se), as calculated for the 1s to 3p levels (K- to M_2,3_-edges) using different TDDFT/TDA schemes.

The computed X-ray absorption and emission spectra of hydrogen selenide (H_2_Se) are reported in Figure 6, considering core levels ranging from 1s to 3p. When studying heavier elements and edges beyond K, complications arise due to the inclusion of contributions from continuum resonances, which are visible as new, potentially very intense features in the L- and M-edge spectra. These resonances primarily arise from the discretized sampling of continuum states associated with excitations from higher-lying edges, e.g., from 2p, 3s, and 3p when considering the 2s edge. For XAS these occur due to valence/outer core transitions to the discretized continuum (see next section); they are present for both TDA and TDDFT but are conveniently removed when using a core–valence separation (CVS).^47,70−73^ The precise position and intensities of these features depend on the molecule and basis set and are generally seen to be more of an issue when probing the outer core regions of heavier elements. For XES negative eigenvalues are probed, and these valence-continuum contributions are thus not present when using TDA but result from (negative) virtual-occupied de-excitations when using full TDDFT.

### Difference between CVS-TDA and TDA

**Figure 7:**
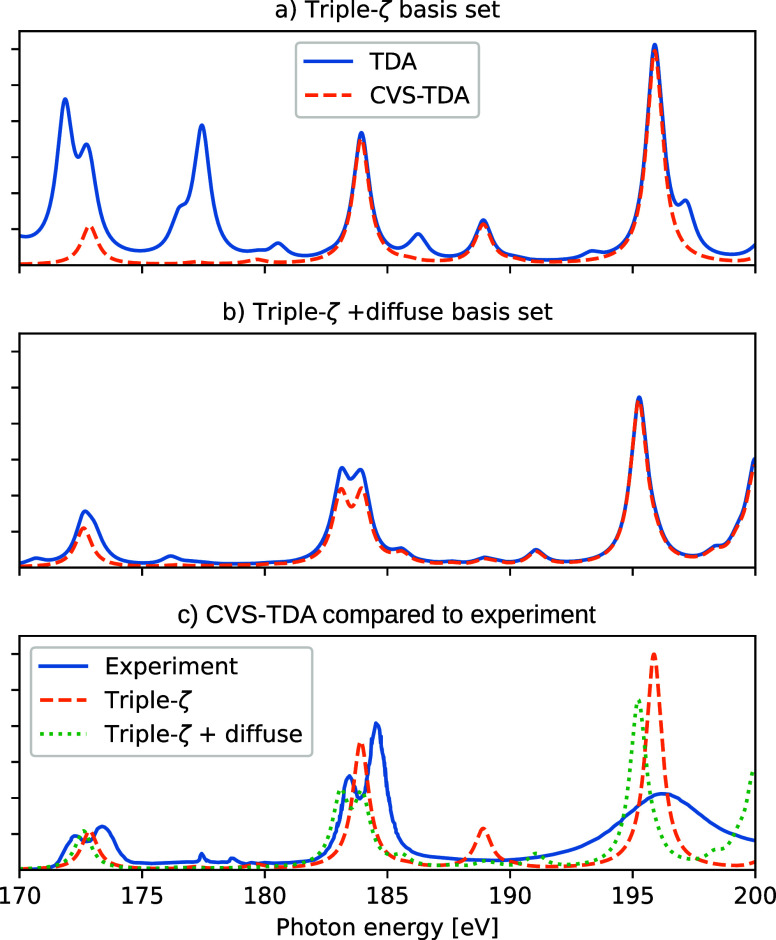
Top panels: Sulfur 2p X-ray absorption spectrum of SF_6_, as calculated using CVS-TDA and full TDA with the B3LYP xc-functional and two different basis sets (see main text for details). Computed spectra have been broadened by a Lorentzian with 0.4 eV HWHM and shifted by 6.5 eV to align with experiment. Bottom panel: Comparing CVS-TDA spectra with experiment.^74^

In order to investigate the influence of the valence-continuum contributions for X-ray absorption spectra, Figure 7 shows the sulfur 3p spectrum of SF_6_, compared to experiment^74^ (extracted using WebPlotDigitizer^75^). This system has previously been studied using real-time TDDFT^70^ and damped linear response theory,^71^ and valence-continuum contributions were noted to be present—CVS-like approaches were then used to remove these contributions. The top two panels of Figure 7 utilize the basis set from ref (70), i.e., aug-cc-pV(T+d)Z for sulfur and a modified aug-cc-pVTZ for fluorine (removing the most diffuse d-function and all f-functions), and the calculations were performed using the B3LYP xc-functional. A larger basis set has been constructed by adding diffuse 19s19p19d functions on sulfur and 10s10p functions on fluorine, here labeled triple-ζ + diffuse, with results shown in the middle panels. The threshold for identifying linear dependence in the basis set was changed to 10^–6^ for these calculations, compared to the PySCF default of 10^–8^. The resulting CVS spectra are in good agreement with the non-relativistic results from Kadek and co-workers,^70^ with the larger basis yielding a split in the feature around 184 eV and the peak around 189 eV being replaced by several, lower-intensity features (which yields better agreement with experiment, as no distinct peak is present in this energy range). A direct comparison to experiment requires spin–orbit coupling, leading to a split of 2p_1/2_ and 2p_3/2_ of about 1.17 eV,^70,74^ which is not present in our calculations.

Comparing the full TDA results to those of CVS-TDA, more intensity is distributed within the shown energy region, in particular for the smaller basis set. These features are a result of the incomplete atom-centered basis set, which yields discretized valence-continuum contributions. Both basis sets yield a number of discretized states within the energy region, but for the larger basis set the corresponding transition dipole moments are distributed over more states (and at different energies) and the effect on the total spectrum is thus dampened. Increasing the basis set size will increase the total number of states, and thus *generally* distribute the intensity over larger regions, but states with high intensity can appear close to a core-resonance. For the TDA results in Figure 7a, the intense extra features are primarily attributed to transitions from fluorine 2s, as can be seen by using CVS space including these MOs (see discussion below). Contributions from continuum states were noted in ref (70) but then primarily at higher energies—this difference is likely due to the use of a 4-component relativistic framework, while our calculations are non-relativistic. As such, the extent of the contributions from the discretized continuum states depends on the molecule, basis set, method, and energy region.

**Figure 8:**
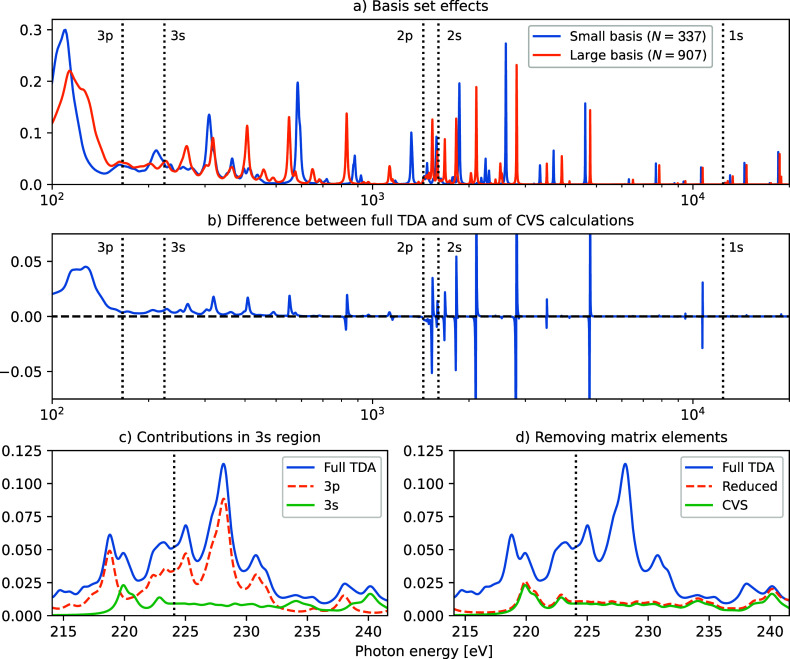
X-ray absorption spectra of hydrogen selenide (H_2_Se), calculated with TDA and CVS-TDA. Features broadened by 5 eV for parts a and b and 0.5 eV for parts c and d. (a) The total spectrum from 0.1 to 20 keV, using two different basis sets (size *N*), as described in the main text. The remaining panels show results obtained with the larger basis set. (b) Difference spectrum between the TDA spectrum and summed CVS-TDA spectra, where each occupied MO is considered in turn. (c) 3s (L_1_) spectrum using full TDA and CVS spaces including 3p and 3s. (d) 3s spectrum using full TDA and 3s CVS space, as well as an approach in which matrix elements are removed if the diagonal element falls within the plotted energy region (save for within the 3s block). Dotted vertical lines indicate the positions of the different ionization energies (estimated using MO energies).

Returning to hydrogen selenide (H_2_Se), Figure 8 shows the effects of using full TDA and different CVS spaces for the selenium 1s- to 3p-edges. Panel a shows the basis set effects on the total spectrum ranging from 0.1 to 20 keV. An uncontracted aug-cc-pVTZ basis set is used with diffuse 25s25p25d basis functions added to the selenium atom. This is then further augmented by adding additional 14s14p14d functions at six ghost atoms positioned 2.5 Å away along the Cartesian axes. The resulting basis sets are of total size 337 and 907, where 115 and 301 basis functions have been removed to avoid linear dependencies. These basis set constructions are inspired by the work of Leetmaa and co-workers,^76^ where the continuum region of the X-ray absorption spectrum of gas phase water was considered by adding many diffuse functions centered at the oxygen atom and at ghost atoms positioned in such a way so as to replicate an ice-like structure. This was seen to yield a very smooth spectrum and was suggested to work well for energies up to 10–15 eV beyond the probed edge. At higher energies, a localized Gaussian-type basis set is likely to be insufficient for describing the higher-lying valence/core-continuum states. Comparing the two spectra in Figure 8a, the larger basis set is seen to yield more smeared out intensities, but there are still very intense features throughout the entire spectrum.

Focusing on the influence of the CVS approximation, panel b shows the difference between the full TDA spectrum and the sum of spectra obtained by using CVS-TDA with each occupied state in the CVS space in turn. This sum of CVS-TDA spectra is expected to yield a poor description for lower energies (on account of the stronger coupling), which is seen from the intense difference between 0.1 keV and the 3p ionization and the generally positive differences stretching up to approximately 1 keV. For higher energies, the difference spectrum primarily consists of features with a positive and negative node, resulting from shifts in the energy position of peaks. For the highest energies, the differences are barely noticeable on the present scale—this is the result both of smaller coupling and of there being few valence- or outer core-continuum resonances at these energies, with the 1s region having only contributions from 1s transitions. These results indicate that CVS is an excellent approximation for excitations from inner core orbitals but yields increasing discrepancies when moving to the outer core region.

Finally, in order to study the origin of the valence-continuum contributions in terms of matrix elements in the TDA Hessian, the bottom two panels of Figure 8 focus on the 3s spectrum region. In panel c the total TDA spectrum is shown, as well as spectra with a CVS space consisting of 3s, and of all 3p. It is seen that most of the additional intensity in the 3s region comes from excitations from 3p, i.e., from the closest (outer) level. The valence-continuum (or outer core-continuum) contributions are associated with diagonal matrix elements in the TDA Hessian with energies close to the diagonal elements in the CVS-block. They can also be investigated by considering the amplitudes of the eigenvectors, an approach that is likely to work better for mixed states. The contributions can then be removed by a number of different methods, such as freezing the contributing virtual MOs, setting the diagonal matrix elements to very high energies (thus shifting the resonance energy), or removing these elements entirely. The latter approach is used in panel d, where a reduced Hessian in which occupied-virtual combinations associated with diagonal matrix elements of an energy within the resolved spectrum region (not counting elements within the CVS space) are removed, thus forming a reduced Hessian. Using this approach, the total Hessian is decreased from size 16,326 to 15,979, which is still significantly larger than the CVS matrix of dimension 907. The resulting spectrum is close to that of CVS, albeit with higher intensities, especially at the edges of the energy region. This is primarily due to the presence of resonances just outside the resolved region, and increasing the energy region in which elements are removed progressively makes the comparison to CVS better and better (the approaches are identical at the limit of an energy region of ±∞).

## Conclusions

The effects of the Tamm–Dancoff approximation (TDA) for calculating X-ray absorption and X-ray emission spectra (XAS and XES) using time-dependent density functional theory (TDDFT) have been investigated. Through explicit diagonalization of the TDDFT/TDA Hessians, the full set of eigenvalues and eigenvectors is obtained, circumventing issues with negative eigenvalues which otherwise plague TDDFT calculations on a core-hole reference state. It is shown that the effects of TDA are limited, primarily amounting to minor changes in intensities. However, for XES and for full-space XAS, valence and outer core-continuum contributions may occur within the energy region of interest, in particular for the outer core region of heavier elements. Using a core–valence separation (CVS) scheme removes these contributions for XAS, as does using TDA for XES. A new CVS-TDA approach for calculating X-ray emission spectra, in which only terms containing the core-hole are retained, leads to a massive decrease in the dimension of the associated eigenvalue equation, while the resulting spectra are seen to be in surprisingly good agreement with the full-space counterparts. However, for most applications we do not see any real reason to use CVS-TDA, as TDA is already relatively inexpensive, only the lowest (negative) eigenvalues are solved, and most working TDDFT implementations have well-tested and optimized TDA solvers.

For XAS, the continuum contributions present when using full TDDFT/TDA have previously been described as artifacts,^70,73^ spurious intruder peaks,^77^ or unphysical valence excitations,^71^ but we note that the use of such terms can potentially be misleading—these features represent an actual coupling to the continuum, but the use of an atom-centered basis set leads to a discretization of this continuum. For energies up to 10–15 eV beyond the ionization threshold the continuum can be described quite well by using very large basis sets (including functions centered at sites outside of the molecule),^76^ but for higher energies this is unlikely to be sufficient. In the limit of a complete basis set (including plane waves of any frequency), the valence/outer core-continuum contributions are expected to yield a correct behavior, featuring a decreasing (but nonzero) absorption cross-section for energies far above the ionization threshold of each absorption edge. Using a Gaussian-type basis set, the discretized resonances can instead be identified by the elements in the Hessian, or the amplitudes in the eigenvectors, and are most conveniently removed using the CVS approximation. This will yield small discrepancies that generally increase as one moves to outer core regions.

In conclusion, for practical calculations, XAS calculations should be performed with a CVS-like approach and either TDDFT or TDA can be used. For XES it is shown that TDA yields stable and reliable spectra, and CVS-TDA is noted to work surprisingly well.
